# Quality and quantity: transitions in antimicrobial gland use for parasite defense

**DOI:** 10.1002/ece3.1827

**Published:** 2015-12-01

**Authors:** Christopher Tranter, Hermógenes Fernández‐Marín, William O. H. Hughes

**Affiliations:** ^1^ School of Life SciencesUniversity of SussexBrightonBN1 9QGUK; ^2^ Instituto de Investigaciones Científicas y Servicios de Alta TecnologíaEdificio 219, Panamá 5Ciudad del SaberClaytonPanama CityPO Box 0843‐01105Republic of Panama

**Keywords:** Attini, disease resistance, fungus‐growing ant, host–parasite evolution, leaf‐cutting ant, metapleural gland

## Abstract

Parasites are a major force in evolution, and understanding how host life history affects parasite pressure and investment in disease resistance is a general problem in evolutionary biology. The threat of disease may be especially strong in social animals, and ants have evolved the unique metapleural gland (MG), which in many taxa produce antimicrobial compounds that have been argued to have been a key to their ecological success. However, the importance of the MG in the disease resistance of individual ants across ant taxa has not been examined directly. We investigate experimentally the importance of the MG for disease resistance in the fungus‐growing ants, a group in which there is interspecific variation in MG size and which has distinct transitions in life history. We find that more derived taxa rely more on the MG for disease resistance than more basal taxa and that there are a series of evolutionary transitions in the quality, quantity, and usage of the MG secretions, which correlate with transitions in life history. These shifts show how even small clades can exhibit substantial transitions in disease resistance investment, demonstrating that host–parasite relationships can be very dynamic and that targeted experimental, as well as large‐scale, comparative studies can be valuable for identifying evolutionary transitions.

## Introduction

Parasites can inflict considerable costs on host organisms (Sheldon and Verhulst 1996; Rigby et al. 2002; Boots et al. 2004). This can result in a dramatic coevolutionary reshaping of the genotype, phenotype and overall life history of both hosts and parasites, and may have been a key influence in major evolutionary transitions including the evolution of sex and sociality (Hamilton et al. 1990; Møller et al. 1993; Boomsma et al. 2005). Understanding how the life history of hosts affects the selection strength on them from parasite pressure, and how this in turn leads to further changes in the evolutionary investment by hosts in costly disease resistance mechanisms, is an important problem in evolutionary biology (Schmid‐Hempel 2001).

Group living is associated with a potential increase in parasite pressure, because a social lifestyle can facilitate the transmission of parasites within the group (Alexander 1974; Cote and Poulin 1995; Møller et al. 2001; Altizer et al. 2003). This is compounded in social insect colonies that have highly homeostatic nest environments and low levels of genetic variation within the colony (Schmid‐Hempel 1994; Frank 1996; Calleri et al. 2006). However, in addition to individual‐level immune defenses, social organisms are able to employ social defenses that have been termed “social immunity” in the broad sense (Dunbar 1991; Cremer et al. 2007; Wisenden et al. 2009; Otti et al. 2014). This can include behavioral defenses such as grooming, and the production and transfer of antimicrobial compounds (Rosengaus et al. 1998, 2004; Fernández‐Marín et al. 2006; Yanagawa et al. 2008; Hamilton et al. 2011; Baracchi et al. 2012; Turnbull et al. 2012), which can mitigate or even outweigh the fitness cost from parasites for group‐living animals (Rosengaus et al. 1998; Hughes et al. 2002; Ugelvig and Cremer 2007; Reber et al. 2011).

The threat of disease has led the most diverse group of social insects, the ants, to evolve a unique exocrine structure, the metapleural gland (MG), which varies in size between species and phenotypes, and in many taxa, produces an antimicrobial secretion that is spread over the cuticle either passively or, in some species, actively by grooming (Hölldobler and Wilson 1990; Bot and Boomsma 1996; Sumner et al. 2003; Fernández‐Marín et al. 2006; Poulsen et al. 2006; Hughes et al. 2008, 2010; Yek and Mueller 2011). Consequently, ants are able to vary their level of investment in disease resistance both on an evolutionary timescale and as a short‐term behavioral response to disease threat by active grooming of the secretion on to the cuticle. While the secretion from the metapleural gland can be antibacterial (Iizuka et al. 1979; Veal et al. 1992), effective at inhibiting fungal sporulation and growth, and helping ants resist parasites (Beattie et al. 1985, 1986; Bot et al. 2002; Graystock and Hughes 2011; Tranter et al. 2014), the gland is energetically costly to maintain (Poulsen et al. 2002b). Thus, its degree of use in different ant species can be used as a measure of their relative investment in disease resistance, and thus, to infer the strength of parasite pressure in different species with different life histories (Hughes et al. 2002; Poulsen et al. 2002a; Boomsma et al. 2005). While experimental blockage of the metapleural gland has been shown to increase the susceptibility of ants to fungal parasites (Poulsen et al. 2002b; Graystock and Hughes 2011; Tranter et al. 2014), the importance of the metapleural gland in disease resistance of individual ants from multiple different species has not previously been quantified directly.

One group of ants which has proved particularly powerful for comparative analyses of disease resistance are the fungus‐growing ants (Attini) that form a monophyletic clade with well‐developed MGs (Table S1; Mueller et al. 2001; Currie et al. 1999; Schultz and Brady 2008; Fernández‐Marín et al. 2013). Fungus‐growing ants have been most extensively investigated with respect to the defense of their fungal crop mutualist against a specialist parasitic fungus, *Escovopsis*. The ants achieve this using a combination of weeding, antimicrobial compounds from the MG, and antimicrobials produced by symbiotic actinomycete bacteria that the ants culture on their cuticles (Currie et al. 1999; Currie 2001a,b; Fernández‐Marín et al. 2013). There appears to be a trade‐off between these two sources of beneficial antimicrobials, with species that possess more of the *Escovopsis*‐specific actinomycete defenses relying less heavily on the MG to protect their fungal crop (Fernández‐Marín et al. 2013). However, while most attention has been focused on the mechanisms by which ants defend their fungal crop against parasites, the ants also need to defend themselves against disease. The antimicrobial compounds produced by the actinomycete bacteria are thought to be specifically active against *Escovopsis* (Currie et al. 1999; 2003; Little et al., 2006; Pagnocca, 2012), and the ants rely for their own defense on mechanisms such as grooming and the MG. The antimicrobial activity of the MG secretion of leaf‐cutting ants has been demonstrated in vitro (Bot et al. 2002), and the importance of the MG for defending both adults and brood against parasites has also been demonstrated in leaf‐cutting ants experimentally (Poulsen et al. 2002a,b; Tranter et al. 2014; ). Previous comparative studies have shown that fungus‐growing ants vary substantially in the size of their MG, the chemical composition of its secretion and their use of active MG grooming to spread the secretion, with the more evolutionary‐derived *Atta* and *Acromyrmex* leaf‐cutting ants that live in larger, more complex societies, having particularly large MG (Fernández‐Marín et al. 2006, 2009, 2013; Hughes et al. 2008; Adams et al. 2012; Vieira et al. 2012b). However, whether these evolutionary transitions in MG size and secretion result in differences in the disease resistance of individual attine ants themselves has not previously directly quantified. In this study, we used MG use as a measure of investment into disease resistance within the evolutionary framework of the fungus‐growing ants. Specifically, we test the hypotheses that when compared to more evolutionarily basal species, the more derived species with larger and more complex societies will show the following: (1) greater reliance on functioning MG glands for their own disease resistance; (2) greater active MG self‐grooming rates in response to a parasite threat; and (3) more powerful antifungal components within the chemical makeup of MG secretions.

## Methods

### Study species

We studied six species of Neotropical attine ants spanning the major phylogenetic divisions within the clade: two species of leaf‐cutting ant (*Atta colombica* and *Acromyrmex echinatior*), three species of higher attines (*Sericomyrmex amabilis*,* Trachymyrmex cornetzi,* and *Trachymyrmex* sp10), and one species of lower attine (*Apterostigma pilosum*). These species represent a spectrum of both the agricultural systems employed within the fungus‐growing ants (from the use of fresh leaves in *Acromyrmex* and *Atta*, to the use of other plant material in *Trachymyrmex* and *Sericomyrmex*, through to the use of general detritus or insect carcasses in *Apterostigma*), as well as the full range of colony sizes, social complexity, and phylogenetic ancestry. Colonies were collected in and around Gamboa, Panama in June 2013, and maintained at 80% relative humidity and 27°C on a 12‐h light/dark cycle. Colonies were fed twice per week with fresh privet (*Ligustrum* sp.) leaves for the two leaf‐cutting species or chopped flower petals and oat flakes for the other four species, and provided with water ad libitum. As the experimental parasite, we used the entomopathogenic fungus *Metarhizium pingshaense* (KVL02‐73; which was originally isolated from soil in Gamboa near a leaf‐cutting ant nest; Hughes et al. 2004). *Metarhizium* is a ubiquitous, generalist fungal pathogen which parasitizes a wide range of insects, including attine and other ants, but which is unlikely because of its generalist nature to have coevolved to overcome the specific defenses of attine ants (Sanchez‐Pena and Thorvilson 1992; Quiroz et al. 1996; Hughes et al. 2004; De Zarzuela et al. 2007; Castilho et al. 2010; Reber and Chapuisat 2011; Ribeiro et al. 2012).

### Experiment 1: the effect of gland blockage and fungal exposure on survival

Twenty worker ants were selected from just inside the nest entrance of each of six colonies of *S. amabilis*,* T. cornetzi, T. *sp10, and *A. pilosum,* 40 ants from six colonies of *A. echinatior,* and 80 ants from three colonies of *A. colombica*. Trials involving *A. echinatior* and *A. colombica* were conducted as two separate cohorts and data later pooled (see Fig. S1). Ants used within each species were a similar size, with medium cuticular coloration, and hence inferred age (Armitage and Boomsma 2010). For the polymorphic leaf‐cutting ant species, we used workers of similar size to the other attines (0.9–1.4 mm head width). Half of the ants from each colony had their MG blocked using quick‐drying nail varnish, while the other half received a control treatment of nail varnish applied to the pronotum. Nail varnish was checked daily and remained intact on all ants treated for the course of the experiment. After 24 h, each of these groups then had either a suspension of *Metarhizium* conidia in 0.05% Triton‐X or a control solution of 0.05% Triton‐X applied topically to the mesosoma with a micropipette. Treatment volumes were standardized for body size between species, and conidia concentrations were approximately the LD_50_ for the species based on pilot studies (5 × 10^6^ conidia per mL for leaf‐cutting ant species and 5 × 10^5^ conidia per mL for the other species; Table S4). This design involved a total of 120 ants for each of *S. amabilis*,* T. cornetzi*,* T. *sp10*,* and *A. pilosum* (30 ants per species for each of the four treatment groups), and 240 ants for *A. colombica* and *A. echinatior* (60 ants per species for each of the four treatment groups). After treatment, each ant was placed in a plastic pot (diameter: 35 mm, height: 70 mm) supplied with cotton balls soaked in 20% sucrose solution and water and kept at 70% relative humidity and 26°C. Ant mortality was recorded for 14 days. Cadavers were immediately removed and surface‐sterilized (Siegel 2012), and then kept in a Petri dish with moistened filter paper for an additional 14 days to allow the sporulation of fungi.

### Experiment 2: the effect of simulated fungal exposure on grooming rates

Twelve ants from each species (two individuals from each of six colonies for *A. echinatior*,* S. amabilis*,* T. cornetzi, T. *sp10, *A. pilosum*, and four individuals from three colonies for *A. colombica*) were observed in a 40‐mm Petri dish for 30 min with two nestmates. Incidences of allogrooming, self‐grooming, and metapleural gland grooming of the focal ant were recorded (Altmann 1974). This process was repeated for 12 additional ants per species, but with each ant receiving a standardized treatment of dry, unscented, talcum powder (magnesium silicate) applied evenly to the dorsal mesosoma and gaster with a fine brush, to induce grooming, prior to observation for 30 min with two nestmates. We used talcum powder because some of the colonies were small and talcum powder particles, which are similar in size to fungal conidia and act as a nonpathogenic stimulant of antiparasite defensive behavior in ants without incurring the mortality that would result from application of fungal conidia (Fernández‐Marín et al. 2006; Morelos‐Juárez et al. 2010; Tranter et al. 2015).

### Experiment 3: chemical inhibition of fungal growth

Six isolated chemical compounds that have been previously identified by Vieira et al. (2012b) as major constituents of attine MG secretions were tested individually for their effect on the conidia viability of the entomopathogenic fungus *Metarhizium pingshaense*. The compounds tested were indole, skatole, methyl oleate (oleic acid), 2‐nonanone, phenylacetic acid, and methyl‐3‐indoleacelate (indoleacetic acid; See Table S2 for percentage compositions found by Vieira et al. 2012b), as well as acetone solvent control, bleach (NaClO) positive control, and ddH_2_O negative control. Each individual compound was tested 10 times at five different concentrations based on the maximal amounts found in the MG of adult *Atta* workers (See Table S4 for summary of dilutions). A conidia solution of 1 × 10^5^ conidia per mL was prepared from freshly sporulating *M. pingshaense* plates. 450 agar plates were prepared with selective media (Sabouraud dextrose agar [SDA] with 0.05 g/L streptomycin sulfate and 0.1 g/L chloramphenicol antibiotics, and 0.1 g/L dodin which inhibits the growth of other fungi but not *Metarhizium*; Shah et al. 2005) in 50‐mm Petri dishes and stored sealed at 4°C until use. 500 μL of the *Metarhizium* conidia solution (5 × 10^6^ conidia per mL) was applied evenly over the surface of the Petri dish and left for 10 min to allow excess liquid to dry. A single, 6‐mm diameter piece of sterile plastic tubing was placed carefully onto the center of the surface of the agar plates, and 20 μL of test solution was applied in the center of this with the plastic cylinder acting as a well to restrict distribution to a defined area. The cylinder was left in place for 5 min to allow the compound to infuse the media, before the location of the treated area was marked on the underside of the Petri dish and the cylinder removed. The Petri dish was then sealed with parafilm and placed in an incubator at 32°C overnight. 12 h later, the percentage of conidia producing a germ tube longer than the conidia diameter (Siegel 2012; conidia viability) was counted for a standardized area (complete area visible in the microscope eyepiece at 400× magnification) within the section where the compound was applied and also an untreated area outside the marked test area, equidistant with the edge of the Petri dish. A further 60 h later, the plates were photographed from above and the average radius of any zone of inhibition, as characterized by an area around the marked test section free from fungal growth, was recorded (Fig. S2).

### Statistical analysis

The effects of *Metarhizium* exposure, gland closure, and ant species, on ant survival in Experiment 1 were analyzed using Cox proportional hazards regression models. Colony‐of‐origin and cohort for the leaf‐cutting ant trials were included in the models to account for the structured nature of the data, but were not statistically significant (*P* > 0.05 in all cases). Pairwise comparisons were made with Kaplan–Meier tests using the Breslow statistic. The numbers of cadavers sporulating with *Metarhizium* for ants with blocked or unblocked glands were examined for each species with Chi‐squared tests. Grooming rates following exposure to talcum powder in Experiment 2 were analyzed using a general linear mixed model with a gamma distribution and log‐link function; colony‐of‐origin was included as a random factor but was not statistically significant (*P* > 0.05 in all cases). Nonsignificant interaction terms were removed stepwise to obtain the minimum adequate model in each case. Pairwise comparisons were conducted between treatments within each species, and between species for each treatment. The effects of compound and dose in Experiment 3 on the size of fungal inhibition zones were analyzed using a generalized linear model with a gamma distribution and log‐link function. Multiple comparisons were controlled for in all analyses using the sequential Bonferroni adjustment. All analyses were performed in IBM SPSS v21 Armonk, NY: IBM Corp.

## Results

### Experiment 1: the effect of gland blockage and fungal exposure on survival

**Figure 1:**
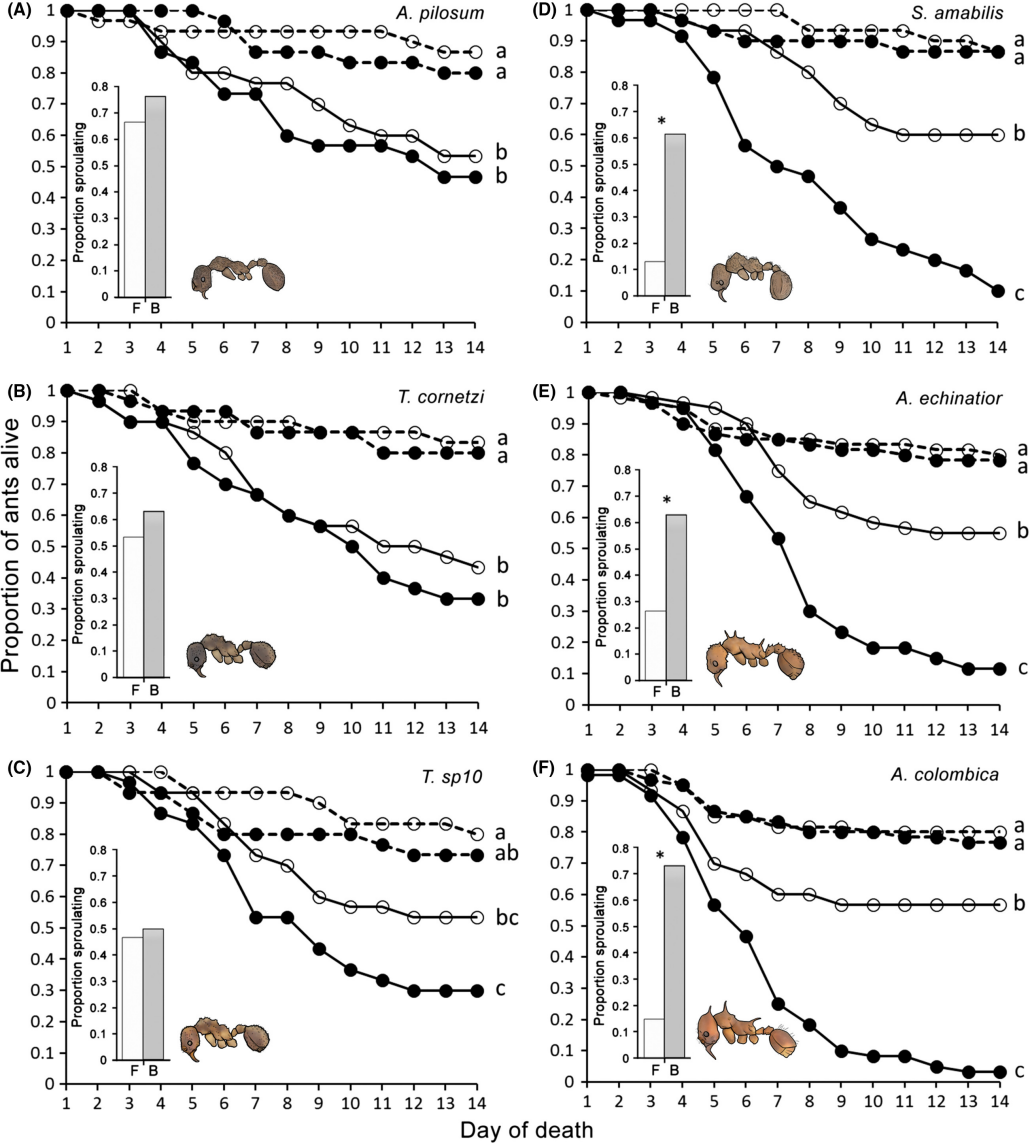
Survival over 2 weeks of (A) *Apterostigma pilosum*, (B) *Trachymyrmex cornetzi*, (C) *Trachymyrmex* sp10, (D) *Sericomyrmex amabilis*, (E) *Acromyrmex echinatior*, and (F) *Atta colombica* attine ants after treatment with either *Metarhizium pingshaense* fungal parasite (solid line) or control solution (dashed line), and with their antimicrobial‐producing metapleural glands either experimentally blocked (closed circles) or functional (open circles). Different letters beside lines indicate treatments which differed significantly from one another within species at *P *<* *0.05. Inset graphs show the proportions of cadavers of *Metarhizium*‐exposed ants that sporulated with *Metarhizium* for ants of each species with either their glands blocked (“B”: dark bars) or functional (“F”: light bars). Species in which the frequency of sporulation differed significantly between ants with blocked and functional glands at *P *<* *0.05 are marked with an asterisk (*).

Overall, there were significant effects of species and interaction between blockage and fungal treatments on survival (*Wald* = 17, df = 5, *P *=* *0.005; *Wald* = 9.98, df = 1, *P *=* *0.02, respectively). Ants from all six species showed a significant reduction in their survival when treated with the *Metarhizium* parasite (Fig. 1; Table S3). *A. colombica*,* A. echinatior,* and *S. amabilis* all showed a significant reduction in resistance to the parasite when their MG was blocked, while the resistance of ants with blocked and unblocked MG was nearly identical in both *T. cornetzi* and *A. pilosum*, and there was also no significant effect of MG blockage on the resistance of *T. *sp10 (Fig. 1; Table S3). There was no significant effect of colony on survival in any of the species (*P *>* *0.05; Table S3). None of the control ants sporulated with *Metarhizium*. Of those *Metarhizium*‐exposed ants that died, significantly more of the cadavers sporulated with *Metarhizium* when they had blocked glands compared to those where the MG was functional (*χ* ^2^ = 47.8, df = 5, *P *<* *0.001). This difference was present in *A. colombica*,* A. echinatior*, and *S. amabilis,* but not in *T. cornetzi*,* T. *sp10, or *A. pilosum* (Fig. 1).

### Experiment 2: the effect of simulated fungal exposure on grooming rates

**Figure 2:**
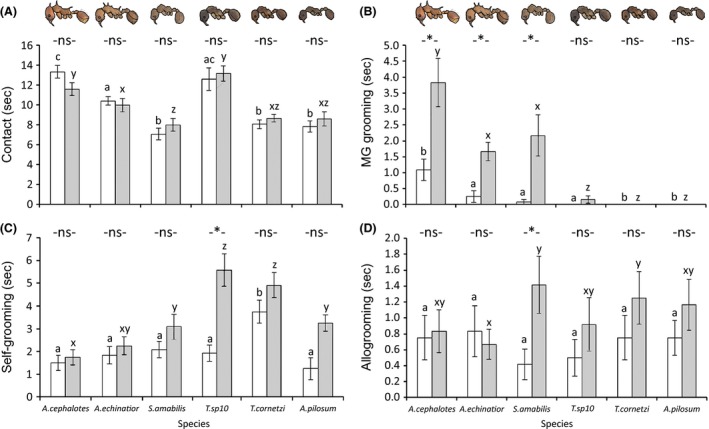
The mean ± SE frequencies of (A) contact, (B) metapleural gland grooming, (C) self‐grooming, and (D) allogrooming in 30 min for six species of attine ants (*Atta colombica*,*Acromyrmex echinatior*,*Sericomyrmex amabilis*,*Trachymyrmex* sp10, *Trachymyrmex cornetzi*, and *Apterostigma pilosum*) either treated with talcum powder (dark bars) or untreated (light bars). Significant differences between treated and untreated ants at *P *<* *0.05 for each species are indicated with an asterisk. Species which differed significantly from one another at *P *<* *0.05 are indicated by different letters, a, b, c for untreated ants, or x, y, z for treatment ants.

Contact rates between ants differed between species (*F*_1,137_ = 20.8, *P *<* *0.001), reflecting interspecific differences in the general activity levels of the ants, but there was no effect of talcum powder application or interaction between effects on activity (*F*_1,132_ = 0.42, *P *=* *0.52; *F*_5,132_ = 1.12, *P *=* *0.36; Fig. 2A). There was, however, a significant interaction between the effects of ant species and talcum powder treatment on MG grooming (*F*_5,132_ = 9.67, *P *=* *0.014). *A. colombica* exhibited the highest rate of MG grooming by far, with *A. echinatior* and *S. amabilis* also conducting higher levels of MG grooming compared to the *Trachymyrmex* and *Apterostigma* species which exhibited little or no MG grooming (Fig. 2B). In spite of the relatively small sample sizes for some species, there was overall also a significant interaction between species and talcum powder treatment on the rates of self‐grooming (*F*_5,132_ = 3, *P *=* *0.014), with *T. *sp10 and *A. pilosum* both self‐grooming significantly more when talcum powder was applied to them and baseline levels of self‐grooming being highest in *T. cornetzi* (Fig. 2C). Only *S. amabilis* allogroomed significantly more when exposed to talcum powder treatment (*F*_1,132_ = 5.5, *P *=* *0.02; Fig. 2D), and there was no overall effect of species or interaction between factors for allogrooming (*F*_5,132_ = 0.4, *P *=* *0.86; *F*_5,132_ = 0.79, *P *=* *0.56).

### Experiment 3: chemical inhibition of fungal growth

**Figure 3:**
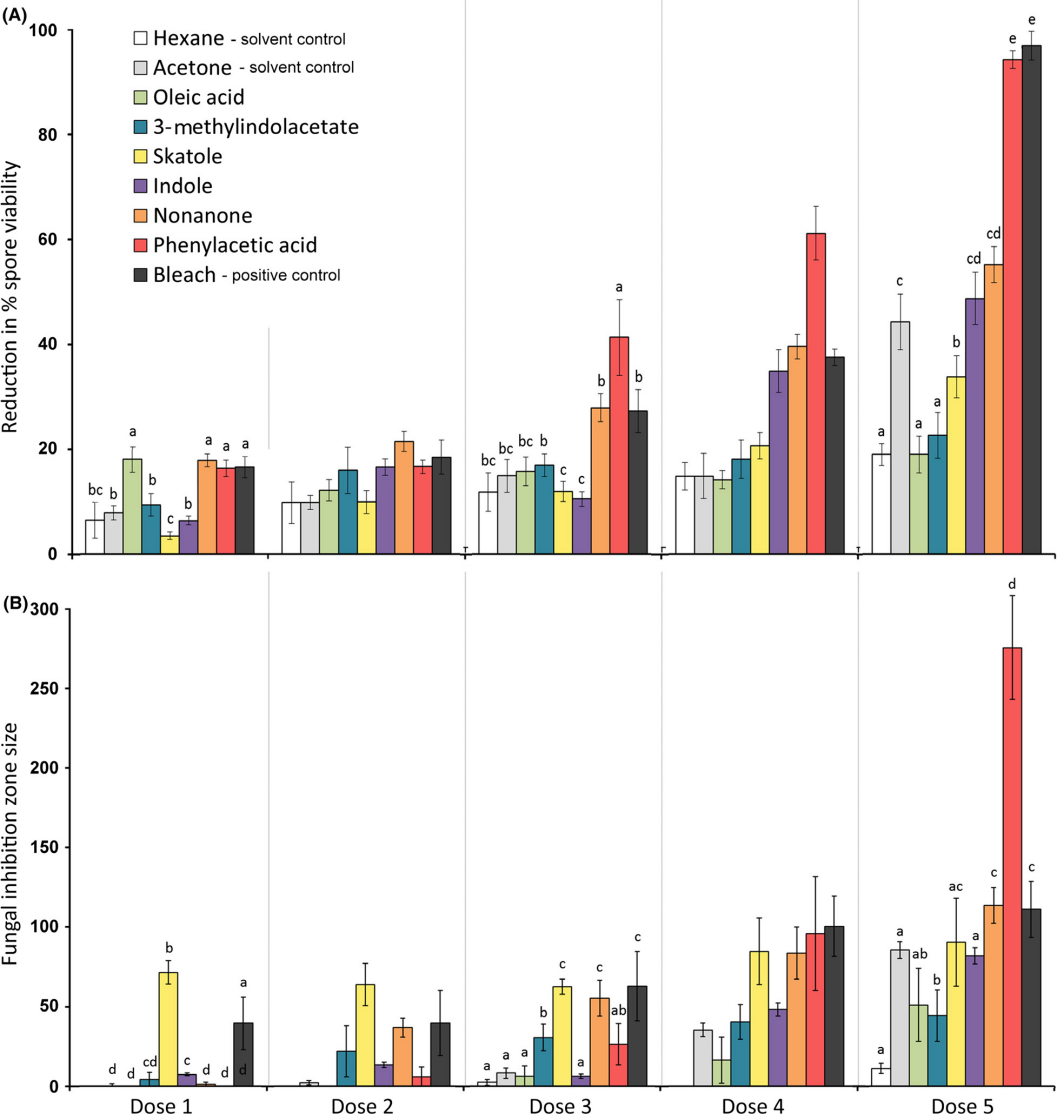
The mean ± SE reduction in viability of conidia of the *Metarhizium pingshaense* fungal parasite (A) and size of growth inhibition zone produced (B), for six chemicals from the metapleural gland secretion (phenylacetic acid, 2‐nonanone, indole, skatole, methyl‐3‐indolacetate, methyl oleate), and bleach positive control, acetone, and hexane solvent control, with each compound applied at five concentrations (1 – lowest dose – 5 highest: see Table S4 for details). A ddH_2_O negative control produced negligible effect and was omitted from the graph. For doses 1, 3, and 5 where pairwise comparisons were performed, different letters indicate chemicals which differed significantly from one another at *P *<* *0.05.

There was a significant interaction between the compound tested and the dose applied on the size of the zone in which *Metarhizium* fungal growth was inhibited (*χ* ^2^ = 194.5, df = 32, *P *<* *0.001), and on the number of fungal conidia that were viable (*χ* ^2^ = 575.4, df = 32, *P *<* *0.001). Phenylacetic acid consistently produced the largest reductions in spore viability, especially at higher doses (Fig. 3A). It also produced the largest inhibition zone in the highest dose, where it was generally comparable to, or even more effective than, bleach in its antifungal activity (Fig. 3B).

## Discussion

Exposure to the fungal parasite *Metarhizium pingshaense* significantly reduced the survival of the six species of ants in this study, and there were species‐specific differences between the resistance of ants when their MGs were blocked, compared with when they were left functional. There were also differences in MG grooming rates and the antifungal activity of chemical components of the MG secretions between species.

**Figure 4:**
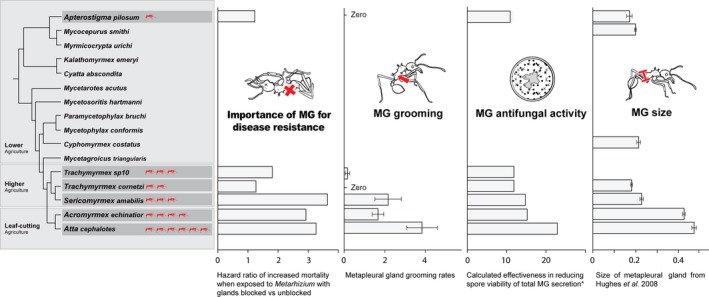
Phylogenetic patterns of metapleural gland usage in the attine ants. Graphs show for the six species studied here (highlighted in dark gray boxes): (1) the hazard ratio from blockage of the antibiotic‐producing metapleural gland for ants exposed to the *Metarhizium* fungal parasite; (2) the mean ± SE frequency of active grooming of the metapleural gland in a 30‐min period; (3) the effectiveness of the overall metapleural gland secretion at reducing *Metarhizium* conidia viability, based on the sum of the average effectiveness of each chemical weighted by its representation in the secretion (Tables S4, S5); (4) the size of the metapleural gland reservoir (bulla width) relative to body size (pronotum width; data from Hughes et al. (2008) and includes data for *Apterostigma collare*, not *Apterostigma pilosum* as in our study). The number of ant symbols shown next to species name represents the typical number of workers per colony in orders of magnitude.

The leaf‐cutting ants *A. colombica* and *A. echinatior*, as well as the higher attine *S. amabilis,* which also has relatively large colony sizes, all demonstrated a consistently greater reliance on MG use for protection against fungal pathogens compared with the more basal *Trachymyrmex* and *Apterostigma* species (Fig. 4). The resistance of leaf‐cutting ants and *Sericomyrmex* to the parasite was significantly reduced when their MG glands were blocked. Additionally, of those ants that died, significantly more of the cadavers sporulated with the parasite when the MG was blocked. This highlights the importance of the gland in sterilizing the ant's cuticle and promoting their survival through fungistatic or fungicidal effects. The leaf‐cutting ants and *S. amabilis* all also showed higher rates of MG grooming behavior, and their secretions contained chemicals with stronger antifungal activity, including more acidic compounds phenylacetic acid and methyl oleate (Do Nascimento and Schoeters 1996; Yek et al. 2012). This was particularly evident in *Atta colombica,* which was the only species in which the highly antifungal phenylacetic acid has been identified (Vieira et al. 2012b; Fernández‐Marín et al. 2015). Additionally, the strength of this effect, and reliance on the MG, seems to be compounded by the efficacy of some of the MG secretions themselves; not only do the leaf‐cutting ants and *S. amabilis* use their glands more actively, but also the compounds in their secretions are more effective antifungal agents. These findings support our predictions that the more derived leaf‐cutting ant species and *S. amabilis* with their larger and more complex societies are considerably more reliant on their MG compared to the more basal species.

The importance of the MG in resisting disease varied across the attine phylogeny providing evidence for the existence of a series of four evolutionary transitions in MG use (Fig. 4). First, between *Apterostigma* and *Trachymyrmex* sp10, we see a small increase in the use of the MG and consequent increased importance of the MG in resisting disease. Second, there is then a small increase in MG size and antifungal activity, and a large increase in MG grooming and the importance of the MG for disease resistance, between *Trachymyrmex* and *Sericomyrmex*. Third, there is a substantial increase in MG gland size between *Sericomyrmex* and the leaf‐cutting ant species. Fourth, there is a transition between *Acromyrmex* and *Atta*, with *Atta* producing a more powerful suite of antifungal chemicals within their MG secretions, which they produce more of and groom much more actively than *Acromyrmex* (Fig. 4). There is no evidence of a trade‐off between different aspects of individual self‐directed MG use, but rather a general trend across the phylogeny toward the MG becoming more effective and important in disease resistance. Although there is a significant phylogenetic distance between *Apterostigma* and *Trachymyrmex*, the differences in MG use between these species were less dramatic than the transitions observed between species higher up the phylogeny, in which there are also more dramatic changes in colony size and complexity. Further studies on more basal species intermediate between *Apterostigma* and *Trachymyrmex* are needed to confirm this and allow formal quantitative phylogenetically controlled analyses, as well as to reveal whether the MG in these species may instead have a role in defense against different microbes.

These differences in MG use, in combination with transitions in other morphological and behavioral attributes such as gardening of the fungal crop or hitchhiking on harvested leaf material, may reflect changes in host–parasite interactions within the attine clade, notably, as a result of specialization of the fungal mutualism and the appearance of polymorphic workers (Hughes et al. 2008; Schultz and Brady 2008; Fernández‐Marín et al. 2009; Griffiths and Hughes 2010; Vieira et al. 2012a). Within the Attini, the leaf‐cutting species, and *Atta* especially, are the most morphologically and behaviorally specialized taxa (Hart et al. 2002; Evison and Ratnieks 2007), and, as we demonstrate here, are especially well adapted for parasite defense. The presence of discreet castes allows for a differentiation in MG morphology in the higher attine species, with the smallest castes having relatively larger MG reservoirs (Hughes et al. 2008). This may aid in parasite resistance through allowing investment into MG defense in those individuals that may benefit from them the most. In leaf‐cutting ants these are the minims that tend to the fungus, clean leaf fragments, and brood (Currie 2001a,b; Hughes et al. 2008; Griffiths and Hughes 2010). It may be that these derived characters have allowed for greater investment into costly disease resistance through improved resource acquisition; for example, the fungal gardens cultivated on fresh vegetation by *Atta* and *Acromyrmex* ants may provide a more nutritious and reliable food source.

Additionally, MG use within the group maps broadly with colony size between the species (Fig. 4; Table S1). Larger colonies have a greater workforce, greater resource acquisition, and are a more robust entity that can provide more stable nest conditions (Kunz 1982; Rosengren et al. 1987; Jeanne and Nordheim 1996; Anderson and Ratnieks 1999; Jones and Oldroyd 2006; Jeanson et al. 2007). However, larger group size may also result in increases in parasite pressure through the same stable and favorable nest conditions and increased colony longevity, which promote parasite transmission (Schmid‐Hempel 1998; Poulin 2007) and may “sample” more of the environment which increases the chance of contracting parasites (Wilson 1971; Rosengren et al. 1987; Sherman et al. 1988; Hölldobler and Wilson 1990; Tschinkel 1991; Schmid‐Hempel 1998; Zahn 1999; Poulin 2007). An alternative explanation is therefore that the larger colonies of leaf‐cutting ants may be exposed to greater parasite pressures than lower attine species and thus invest more in disease resistance in order to mitigate this increased cost (Hart and Ratnieks 1998; Hart and Ratnieks 2001; Hughes et al. 2002; Naug and Camazine 2002; Poulsen et al. 2002b; Fernández‐Marín et al. 2006, 2009; Tranter et al. 2014).

Our results show how changes in the antifungal activity and use of MG secretions affect the resistance of individual ants to parasites, and demonstrate that even relatively small clades can exhibit substantial transitions in investment into disease resistance mechanisms. This highlights how dynamic the evolutionary relationships between host and parasite can be, and demonstrates the value of targeted experimental studies on multiple species for identifying and understanding evolutionary transitions in host–parasite relationships.

## Conflict of Interest

This work was supported by the Biotechnology and Biological Sciences Research Council. The authors of this paper declare no conflict of interests. All applicable international, national, and institutional guidelines for the care and use of animals were followed. This article does not contain any studies with human participants performed by any of the authors.

## Supporting information


**Figure S1**. Experimental groups and species cohort information for Exp 1.

**Figure S2**. Photos (top) of two plates (left: phenylacetic acid, right: bleach) showing zones around central application point in which growth of the Metarhizium fungal parasite was inhibited.

**Table S1**. Fungus growing ant life‐history traits.
**Table S2.** MG compounds tested as a percentage of total secretion volume for Attine genera.
**Table S3.** Statistical results of survival analysis in six attine species with blocked or functional metapleural glands, and treated with *Metarhizium pinshaense* fungal parasite or control solution.
**Table S4**. Isolated MG secretions listed by their abundances as reported in GC‐MS fractions (Do Nascimento and Schoeters 1996; Ortius‐Lechner et al. 2000; Vieira et al. 2012b) and natural gland secretions from *Acromyrmex* ants.
**Table S5**. The natural abundances, individual antimicrobial activities, and calculated percentage antimicrobial activity for MG secretion compounds tested in Attine ants.

**Table S6**. Experiment 1: Survival of ants treated with Triton‐X control or Metarhizium fungus with glands blocked or unblocked.
