# Supplementary figures and images for: Quality and quantity: transitions in antimicrobial gland use for parasite defense
Source: Ecol Evol. 2015 Dec 1;5(24):5857–68. doi: 10.1002/ece3.1827 (PMC4717345; doi:10.1002/ece3.1827)

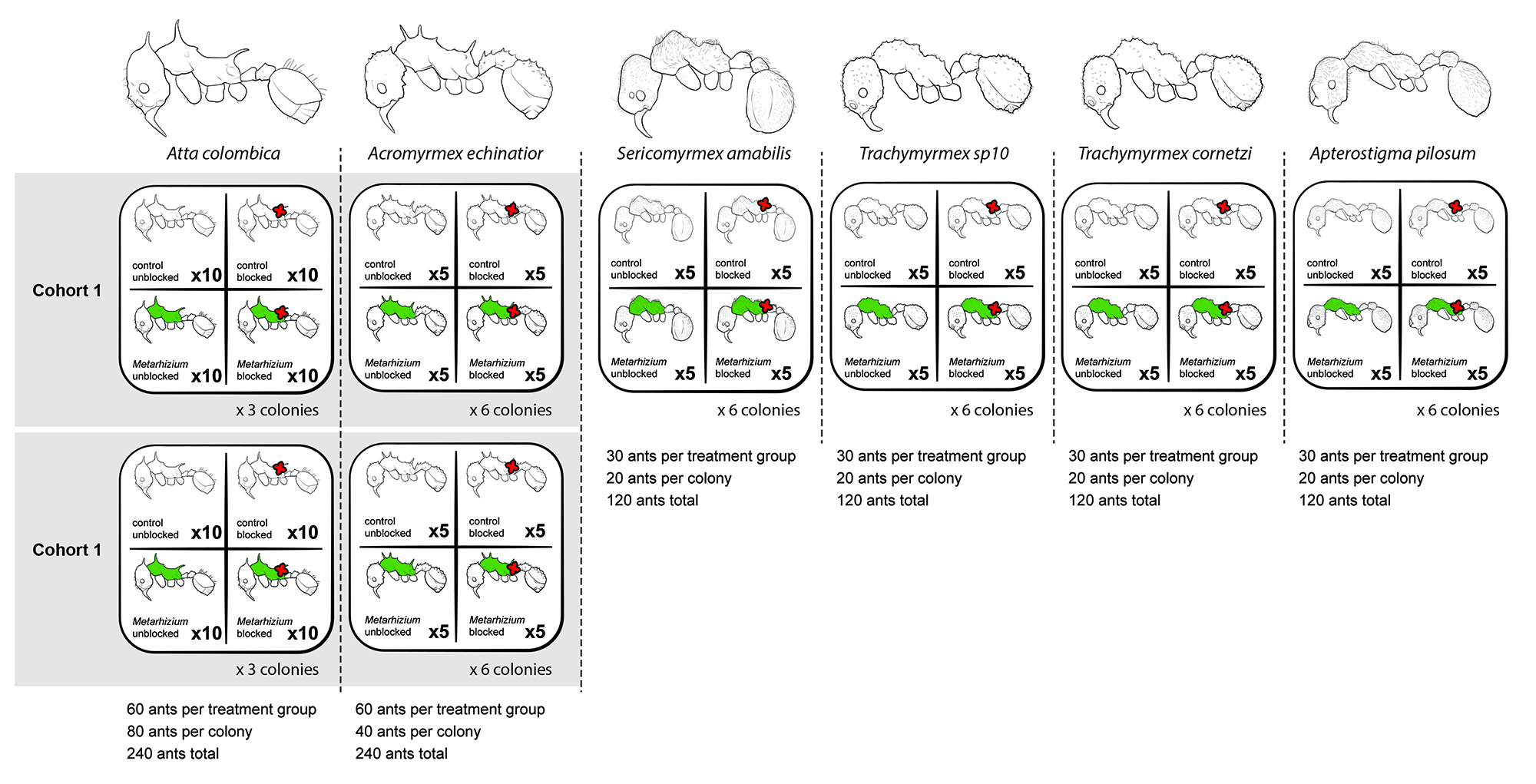

Supplement: Supplementary file 1 — Figure S1. Experimental groups and species cohort information for Exp 1. [file ECE3-5-5857-s001.tif]

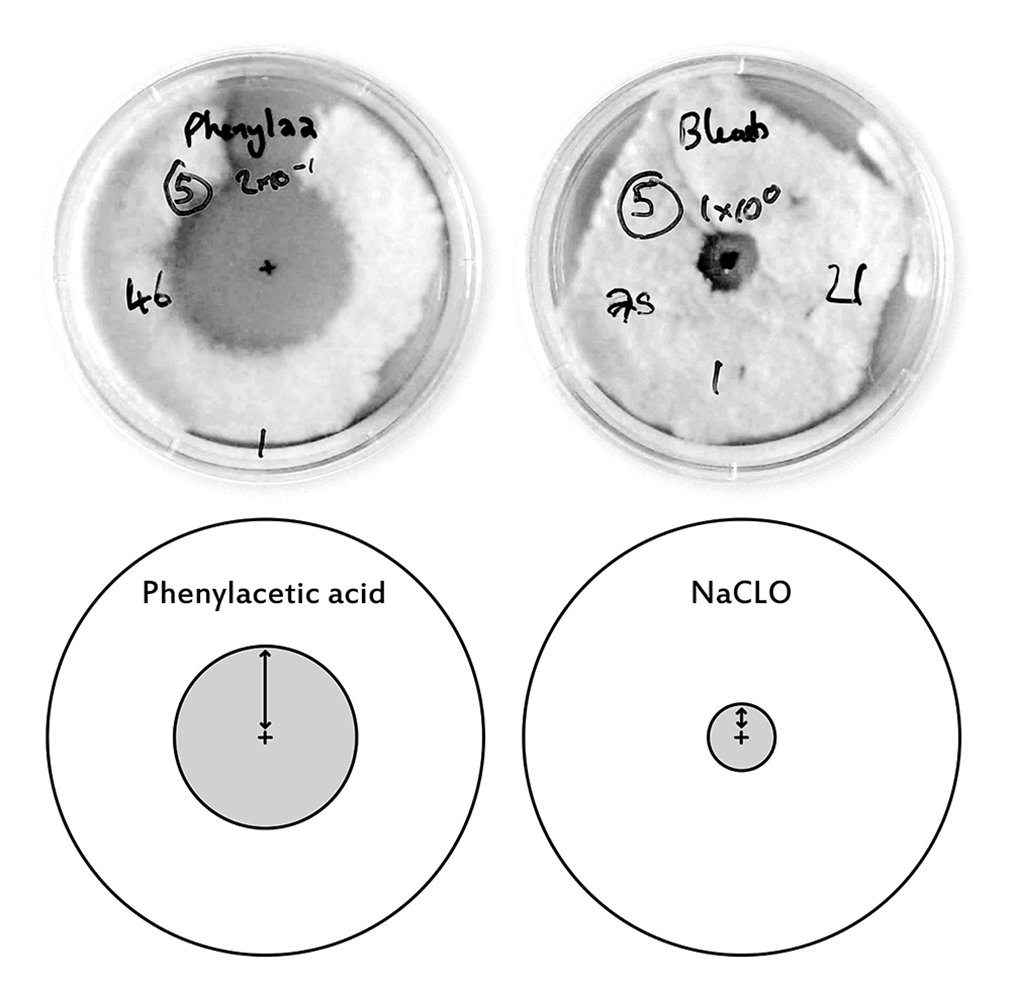

Supplement: Supplementary file 2 — Figure S2. Photos (top) of two plates (left: phenylacetic acid, right: bleach) showing zones around central application point in which growth of the Metarhizium fungal parasite was inhibited. [file ECE3-5-5857-s002.tif]
