# Supplementary material for: Quality and quantity: transitions in antimicrobial gland use for parasite defense
Source: Ecol Evol. 2015 Dec 1;5(24):5857–68. doi: 10.1002/ece3.1827 (PMC4717345; doi:10.1002/ece3.1827)
Supplement: Supplementary file 3 — Table S1. Fungus growing ant life‐history traits. Table S2. MG compounds tested as a percentage of total secretion volume for Attine genera. Table S3. Statistical results of survival analysis in six attine species with blocked or functional metapleural glands, and treated with Metarhizium pinshaense fungal parasite or control solution. Table S4. Isolated MG secretions listed by their abundances as reported in GC‐MS fractions (Do Nascimento and Schoeters 1996; Ortius‐Lechner et al. 2000; Vieira et al. 2012b) and natural gland secretions from Acromyrmex ants. Table S5. The natural abundances, individual antimicrobial activities, and calculated percentage antimicrobial activity for MG secretion compounds tested in Attine ants. [file ECE3-5-5857-s003.docx]

**Supplementary Material**

| **Table S1**. Fungus growing ant life-history traits | | | | | | | | | | |
| --- | --- | --- | --- | --- | --- | --- | --- | --- | --- | --- |
|  | | Colony size | | Life history summary | | MG grooming rates | | Antibiotic-producing actinomycete coverage | |  |
| *Atta  colombica* | Very large  10^6^ | | Polymorphic. Huge underground colonies. Cut leaves as fungus substrate  ‘Leafcutter group’ | | Very high | | None | |  |  |
| *Acromyrmex echinatior* | Large  10^4^ | | Polymorphic. Cut leaves as fungal substrate  ‘Leafcutter group’ | | High | | Low | |  |  |
| *Sericomyrmex amabilis* | Medium  10^3^ | | Monomorphic. Multiple fungal chambers.  ‘Higher agriculture’ | | High | | Low | |  |  |
| *Trachymyrmex cornetzi* | Small  ca. 10^2^ | | Monomorphic  ‘Higher agriculture’ | | Very low | | High | |  |  |
| *Trachymyrmex sp10* | Medium  10^3^ | | Monomorphic  ‘Higher agriculture’ | | Low | | None | |  |  |
| *Apterostigma pilosum* | Very small  10^1^ | | Monomorphic. Dead plant or arthropod material as substrate. ‘Coral fungus group’ | | Very low | | Low | |  |  |

References: (Weber 1972; Hölldobler and Wilson 1990; Murakami *et al*., 2000; Mikheyev *et al*., 2007; Pitts-Singer and Espelie 2007; Baer *et al*., 2009; Fernández-Marín *et al*., 2009, 2013; Mehdiabadi and Schultz 2010; Leal *et al*., 2011; Mueller *et al*., 2011; Bruner *et al*., 2013)

| **Table S2.** MG compounds tested as a percentage of total secretion volume for Attine genera. Modified from Vieira *et al*., 2006. Product source identifier in brackets (Sigma-Aldrich); n.d. = not detectable. | | | | | | | | |
| --- | --- | --- | --- | --- | --- | --- | --- | --- |
|  | | | % chemical constituent of MG secretion | | | | | |
|  |  |  | Indole  ([I3408](http://www.sigmaaldrich.com/catalog/product/aldrich/i3408?lang=en&region=GB)) | Skatole  (M51458) | Methyl oleate  (311111) | Methyl-3-indoleacelate  (I9770) | 2-Nonanone  (W278505) | Phenylacetic acid  (P16621) |
| Attine species |  | *Apterostigma pilosum* | 1.4 | 95.5 | n.d | 2.3 | n.d | n.d |
|  |  | *Mycetarotes parallelus* | 13.1 | 55.9 | 15.5 | n.d | n.d | n.d |
|  |  | *Trachymyrmex fuscus* | 7.9 | 68.5 | 1.1 | 10.9 | n.d | n.d |
|  |  | *Acromyrmex coronatus* | n.d | 40.9 | 1 | 2.8 | 11.7 | n.d |
|  |  | *Atta laevigata* | 8.7 | 36.2 | 0.5 | 5.1 | 6.1 | 29.3 |

| **Table S3.** Statistical results of survival analysis in six attine species with blocked or functional metapleural glands, and treated with *Metarhizium pinshaense* fungal parasite or control solution. | | | |
| --- | --- | --- | --- |
| Ant species | Factor | Wald | Significance |
| *Atta colombica* | Gland blockage  Fungal treatment  Blockage * Fungal  Colony | 22.04  8.27  4.36  0.37 | <0.001*  0.004*  0.037*  0.841 |
| *Acromyrmex echinatior* | Gland blockage  Fungal treatment  Blockage * Fungal  Colony | 18.35  6.96  3.92  0.58 | <0.001*  0.008*  0.048*  0.989 |
| *Sericomyrmex amabilis* | Gland blockage  Fungal treatment  Blockage * Fungal  Colony | 28.60  10.55  5.01  10.56 | <0.001*  0.001*  0.025*  0.061 |
| *Trachymyrmex sp10* | Gland blockage  Fungal treatment  Blockage * Fungal  Colony | 2.94  4.36  0.10  1.73 | 0.087  0.035*  0.75  0.885 |
| *Trachymyrmex cornetzi* | Gland blockage  Fungal treatment  Blockage * Fungal  Colony | 1.32  9.80  0.07  12.08 | 0.25  0.002*  0.79  0.06 |
| *Apterostigma pilosum* | Gland blockage  Fungal treatment  Blockage * Fungal  Colony | 0.63  5.85  0.01  8.65 | 0.43  0.016*  0.92  0.124 |

| **Table S4**. Isolated MG secretions listed by their abundances as reported in GC-MS fractions (Do Nascimento and Schoeters 1996; Ortius-Lechner *et al*., 2000; Vieira *et al*., 2012b) and natural gland secretions from *Acromyrmex* ants. Total secretion volumes taken as an average of 4µL (Ortius-Lechner *et al*., 2000; Yek *et al*., 2012). Calculated dilution series based on natural concentrations and appropriate solvent are also listed. Concentration 3 in bold is representative of the calculated natural concentration thought to be found in ants. Compounds for each dilution were first dissolved in their appropriate solvent and then further diluted with ddH20 to reach the correct concentration of test compound and a concentration of solvent which matches the appropriate dilution of control solvent. E.g. 2.4g of indole were dissolved in 0.5 mL of acetone, which was then added to 0.5 mL of ddH­_2_O. | | | | | | | | | | |
| --- | --- | --- | --- | --- | --- | --- | --- | --- | --- | --- |
| MG secretion test compounds | Amount found in GC-MS analysis (ng) | | Concentration in ant MG secretion (ng/µL) | Concentrations tested (g/mL) | | | | | Solvent used (see below) | Volume solution applied (µL) |
|  |  |  |  | 1  1x10^-2^ | 2  1x10^-1^ | 3  1x10^0^ | 4  1x10^1^ | 5  1x10^2^ |  |  |
| Indole | 95 | | 24 | 2.4 x10^-4^ | 2.4 x10^-3^ | 2.4 x10^-2^ | 2.4 x10^-1^ | 2.4 x10^0^ | Acetone | 20 |
| Skatole | 430 | | 108 | 1.1 x10^-3^ | 1.1 x10^-2^ | 1.1 x10^-1^ | 1.1 x10^0^ | 1.1 x10^1^ | Acetone | 20 |
| Methyl oleate | 6 | | 1.5 | 1.5 x10^-5^ | 1.5 x10^-4^ | 1.5 x10^-3^ | 1.5 x10^-2^ | 1.5 x10^-1^ | Acetone | 20 |
| 2-Nonanone | 30 | | 7.5 | 7.5 x10^-5^ | 7.5 x10^-4^ | 7.5 x10^-3^ | 7.5 x10^-2^ | 1.2 x10^-1^ | Hexane | 20 |
| Phenylacetic acid | 350 | | 87.5 | 8.8 x10^-4^ | 8.8 x10^-3^ | 8.8 x10^-2^ | 8.8 x10^-1^ | 8.8 x10^0^ | ddH_2_0 | 20 |
| Methyl-3-indoleacetate | 40 | | 10 | 1.0 x10^-4^ | 1.0 x10^-3^ | 1.0 x10^-2^ | 1.0 x10^-1^ | 1.0 x10^0^ | Acetone | 20 |
|  |  | |  |  |  |  |  |  |  |  |
| Solvent control chemical | Control type | |  | Dilutions tested | | | | | Diluted in |  |
|  |  |  |  | 1 | 2 | 3 | 4 | 5 |  |  |
| Hexane | solvent control | |  | 5 x10^-5^ | 5 x10^-4^ | 5 x10^-3^ | 5 x10^-2^ | 5 x10^-1^ | ddH_2_0 |  |
| Acetone | solvent control | |  | 5 x10^-5^ | 5 x10^-4^ | 5 x10^-3^ | 5 x10^-2^ | 5 x10^-1^ | ddH_2_0 |  |
| Bleach | positive control | |  | 5 x10^-5^ | 5 x10^-4^ | 5 x10^-3^ | 5 x10^-2^ | 5 x10^-1^ | ddH_2_0 |  |
| ddH_2_0 | negative control | |  | 1 | 1 | 1 | 1 | 1 | n/a |  |
| Dose volumes applied standardised for body size | |  | | |  |  |  |  |  |  |
|  |  |  |  |  |  |  |  |  |  |  |
| *Atta, Acromyrmex*  *Sericomyrmex, Trachymyrmex sp10*  *Trachymyrmex cornetzi, Apterostigma* | | 0.5 µl  0.4 µl  0.3 µl | | |  |  |  |  |  |  |

**Table S5.**

| **Natural abundances** |  |  |  |  |  |  |  |
| --- | --- | --- | --- | --- | --- | --- | --- |
| ***Species*** | ***Indole*** | ***Skatole*** | ***Methyl oleate*** | ***Methyl-3-indol.*** | ***2-Nonanone*** | ***Phenylacetic acid*** | ***total*** |
| *A.cephalotes* | 8.70 | 36.20 | 0.50 | 5.10 | 6.10 | 29.30 | 85.90 |
| *A.echinatior* | 0.00 | 40.90 | 1.00 | 2.80 | 11.70 | 0.00 | 56.40 |
| *S.amabilis* | 2.45 | 50.71 |  | 6.61 | 14.00 |  | 73.77 |
| *T.sp10* | 7.90 | 68.50 | 1.10 | 10.90 | 1.10 | 0.00 | 89.50 |
| *T.cornetzi* | 3.90 | 84.11 | 0.80 | 10.90 | 0.00 | 0.00 | 99.71 |
| *A.pilosum* | 1.40 | 95.50 | 0.00 | 2.30 | 0.00 | 0.00 | 99.20 |
|  |  |  |  |  |  |  |  |
| **Compound average antimicrobial activities (Fig 3a)** | | | |  |  |  |  |
|  | ***Indole*** | ***Skatole*** | ***Methyl oleate*** | ***Methyl-3-indol.*** | ***2-Nonanone*** | ***Phenylacetic acid*** | ***total*** |
|  | 10.45 | 11.88 | 15.70 | 10.45 | 27.87 | 41.29 |  |
|  |  |  |  |  |  |  |  |
| **Percentage composition** |  |  |  |  |  |  |  |
| ***Species*** | ***Indole*** | ***Skatole*** | ***Methyl oleate*** | ***Methyl-3-indol.*** | ***2-Nonanone*** | ***Phenylacetic acid*** | ***total*** |
| *A.cephalotes* | 10.13 | 42.14 | 0.58 | 5.94 | 7.10 | 34.11 | 100.00 |
| *A.echinatior* | 0.00 | 72.52 | 1.77 | 4.96 | 20.74 | 0.00 | 100.00 |
| *S.amabilis* | 3.32 | 68.74 | 0.00 | 8.96 | 18.98 | 0.00 | 100.00 |
| *T.sp10* | 8.83 | 76.54 | 1.23 | 12.18 | 1.23 | 0.00 | 100.00 |
| *T.cornetzi* | 3.91 | 84.35 | 0.80 | 10.93 | 0.00 | 0.00 | 100.00 |
| *A.pilosum* | 1.41 | 96.27 | 0.00 | 2.32 | 0.00 | 0.00 | 100.00 |
|  |  |  |  |  |  |  |  |
| **Antimicrobial activities x Percentage composition** | | |  |  |  |  |  |
| ***Species*** | ***Indole*** | ***Skatole*** | ***Methyl oleate*** | ***Methyl-3-indol.*** | ***2-Nonanone*** | ***Phenylacetic acid*** | ***total*** |
| *A.cephalotes* | 1.06 | 5.01 | 0.09 | 0.62 | 1.98 | 14.08 | 22.84 |
| *A.echinatior* | 0.00 | 8.61 | 0.28 | 0.52 | 5.78 | 0.00 | 15.19 |
| *S.amabilis* | 0.35 | 8.17 | 0.00 | 0.94 | 5.29 | 0.00 | 14.74 |
| *T.sp10* | 0.92 | 9.09 | 0.19 | 1.27 | 0.34 | 0.00 | 11.82 |
| *T.cornetzi* | 0.41 | 10.02 | 0.13 | 1.14 | 0.00 | 0.00 | 11.70 |
| *A.pilosum* | 0.15 | 11.44 | 0.00 | 0.24 | 0.00 | 0.00 | 11.83 |
